# Checklist of the tidal pool fishes of Jeju Island, Korea

**DOI:** 10.3897/zookeys.709.14711

**Published:** 2017-10-18

**Authors:** Hyuck Joon Kwun, Jinsoon Park, Hye Seon Kim, Ju-Hee Kim, Hyo-Seon Park

**Affiliations:** 1 National Marine Biodiversity Institute of Korea, 75, 101 Jangsan-ro, Janghang-eup, Seocheon-gun, Chungcheongnam-do 33662, Korea; 2 Korea Maritime and Ocean University, 727 Taejong-ro, Yeongdo-gu, Busan 49112, Korea

**Keywords:** coastal habitats, fish diversity, inventory, northwestern Pacific

## Abstract

Seventy-six species of fishes, representing 60 genera and 34 families, were recorded from tidal pools on Jeju Island, southern Korea. The major families in terms of species were the Gobiidae (11 species), Pomacentridae (8 species), Blenniidae (6 species), and Labridae (5 species). Thirty-nine species were classified as tropical, 26 as temperate and 11 as subtropical.

## Introduction

Jeju Island is located southwest of the Korean Peninsula (Kim and Go 2003, Kim et al. 2009, Kwun et al. 2017) and has a volcanic rocky shoreline. The island lies in the southernmost temperate region of Korea, and many subtropical and temperate species of fishes inhabit the coastal and adjacent waters of the island (Kim 2009). Many marine fish species use tidal pools for part of their life cycle (Andrade et al. 2007, González-Murcia et al. 2012, Choi and Lee 2013), so studies of the occurrence of fish species in tidal pools provide important information on local fish biodiversity and for the conservation of coastal ecosystems (Machado et al. 2015, Torres-Hernández et al. 2016).

The first checklist of the fishes of Jeju Island (Uchida and Yabe 1939) included 162 species from coastal and adjacent waters. Subsequent inventories (Kim and Lee 1994, Yoo et al. 1995, Kim et al. 2009) recorded up to 655 species from the island environs, including freshwater fishes, and additional species have more recently been reported from the island’s coastal waters (Kwun et al. 2012, Kwun et al. 2013, Song et al. 2013, Kim et al. 2014, Myoung et al. 2014, Kim 2015, Lee and Kim 2015, Kwun et al. 2016b, Yeo and Kim 2016).

Until now, Kwun et al. (2017) provided the only report specifically addressing fishes from tidal pools of the island. Their preliminary sampling of tidal pools in four regions of the island yielded 50 species for which voucher specimens were retained. However, the sampling only encompassed four months (July–October) and therefore did not account for seasonal occurrences of tidal pool fishes.

Here the first comprehensive list of tidal pool fishes on Jeju Island is provided, based on a detailed survey over all four seasons.

## Materials and methods

**Figure 1.**
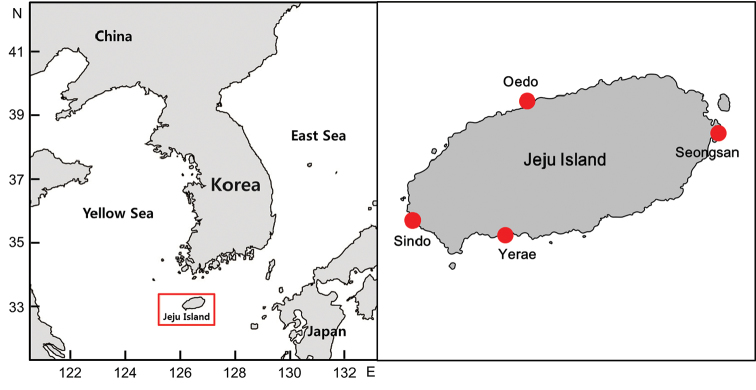
Map showing the location of Jeju Island and sampling sites.

**Figure 2.**
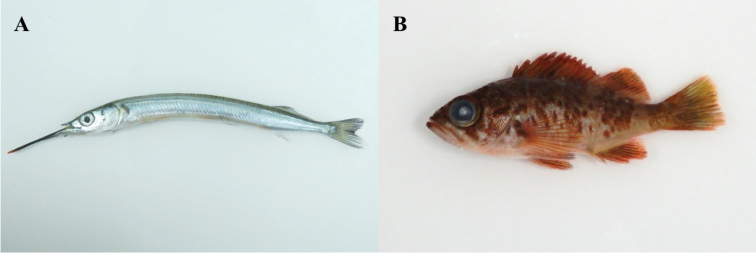
**A** *Hyporhamphus sajori*, MFD-746, 77.3 mm standard length (SL) **B** *Sebastes inermis*, MFD-902, 47.5 mm SL.

**Figure 3.**
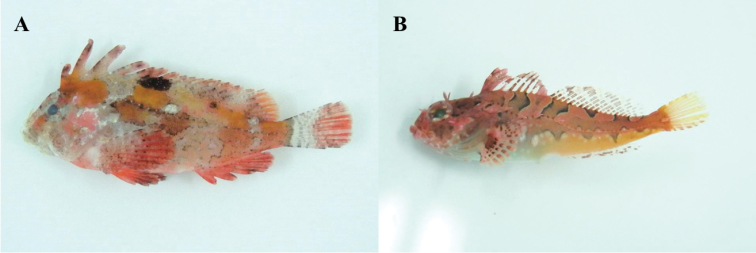
**A** *Hypodytes rubripinnis*, MFD-669, 42.9 mm SL **B** *Furcina osimae*, MFD-627, 29.3 mm SL.

**Figure 4.**
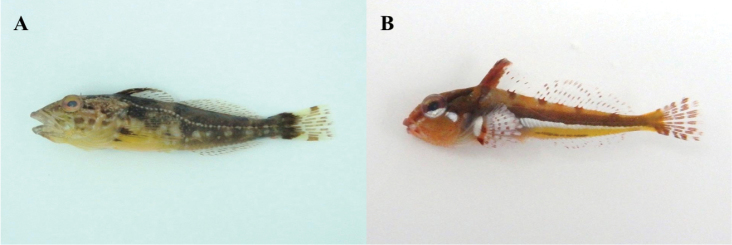
**A** *Pseublennius percoides*, MFD-671, 47.8 mm SL **B** *Pseudoblennius marmoratus*, MFD-927, 19.3 mm SL.

**Figure 5.**
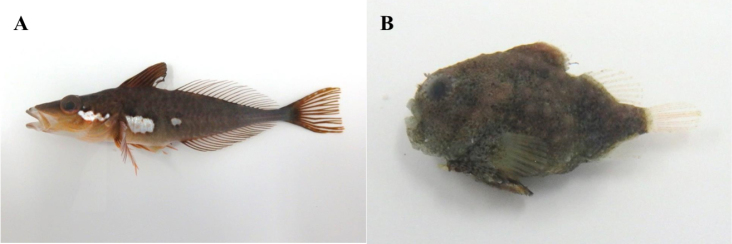
**A** *Vellitor centropomus*, MFD-933, 75.6 mm SL **B** *Eumicrotremus uenoi*, MFD-932, 17.8 mm SL.

**Figure 6.**
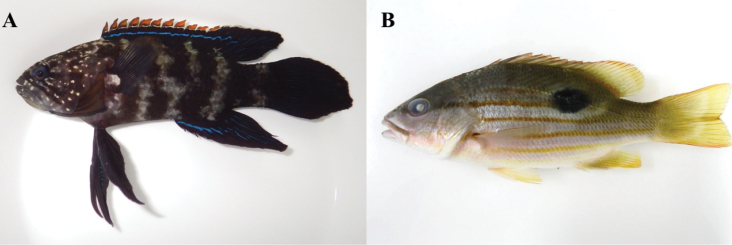
**A** *Plesiops coeruleolineatus*, MFD-860, 82.8 mm SL **B** *Lutjanus fulviflamma*, MFD-866, 101.1 mm SL.

**Figure 7.**
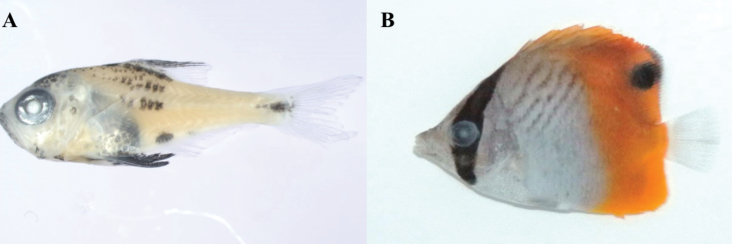
**A** *Pempheris japonica*, MFD-894, 10.8 mm SL **B** *Chaetodon auriga*, MFD-778, 25.7 mm SL.

**Figure 8.**
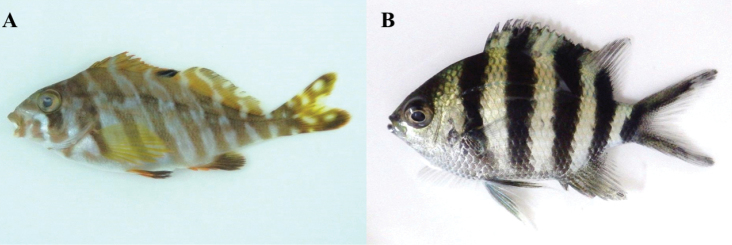
**A** *Goniistius zonatus*, MFD-663, 55.6 mm SL **B** *Abudefduf sexfasciatus*, MFD-845, 33.7 mm SL.

**Figure 9.**
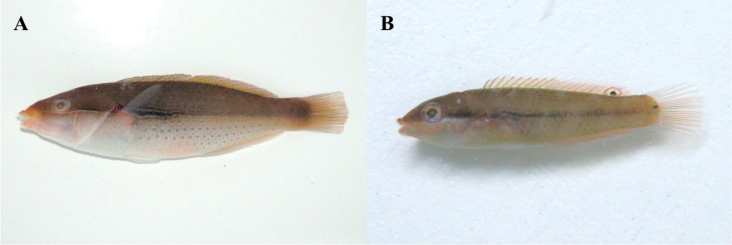
**A** *Stethojulis interrupta terina*, MFD-850, 92.9 mm SL **B** *Stethojulis trilineata*, MFD-482, 25.7 mm SL.

**Figure 10.**
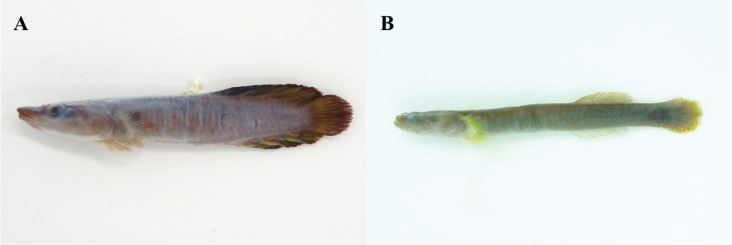
**A** *Lepadichthys frenatus*, MFD-318, 47.5 mm SL **B** *Luciogobius guttatus*, MFD-144, 50.2 mm SL.

**Figure 11.**
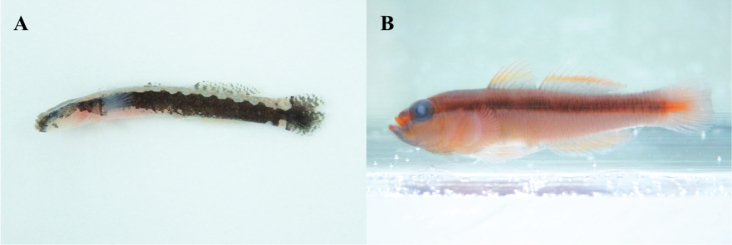
**A** *Clariger cosmurus*, MFD-682, 29.8 mm SL **B** *Trimma grammistes*, MFD-495, 20.7 mm SL.

**Figure 12.**
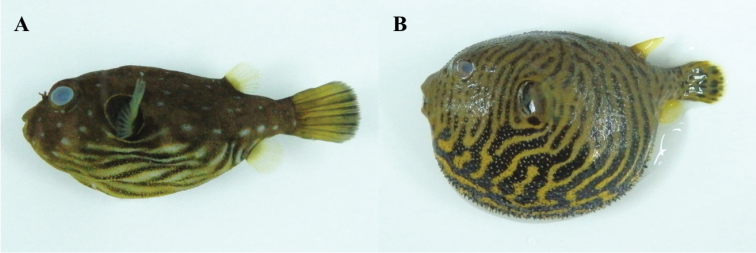
**A** *Arothron hispidus*, MFD-807, 36.2 mm SL **B** *Arothron stellatus*, MFD-808, 53.1 mm SL.

The study area involved four coastal regions of Jeju Island, including areas where Kwun et al. (2017) collected fishes: Oedo (north), Sindo (west), Yerae (south), and Seongsan (east) (Fig. 1). Fishes were caught using hand net and dredge, and all specimens were fixed whole in 99% ethanol. Our fish list was based on the data of Kwun et al. (2017), but many fishes caught during the survey, which occurred between February 2016 and January 2017 (all four seasons), were new records for the four regions. The specimens were classified to family level according to An (2011), and species were identified according to Kim et al. (2005), Allen and Erdmann (2012), Nakabo (2013), Allen et al. (2015), Ikeda and Nakabo (2015), and Kwun and Kim (2015). Fishes were categorized by climate zone on group (temperate, subtropical, and tropical) according to Froese and Pauly (2017). Photographs of the newly collected fishes are provided in Figures 2–12, and the voucher specimen list is provided as an Appendix. Voucher specimens were deposited at the National Marine Biodiversity Institute of Korea, Marine Fish Diversity (MFD; formerly Biodiversity Dynamics Team: BDT).

## Results and discussion

Seventy-six fish species, representing 60 genera and 34 families, were recorded (Table 1). The sample from Seongsan included the largest number of species (54), followed by Yerae (44 species), Sindo (38 species), and Oedo (31 species) (Table 2). The most important families in terms of species were the Gobiidae (11 species), followed by Pomacentridae (8), Blenniidae (6) and Labridae (5). Among the 76 species, 20 occurred in all four regions. The tidal pools of Jeju Island have the highest fish species diversity in such habitats yet reported worldwide, exceeding the 72 species reported from Yakushima Island, Japan, and 62–63 species reported from Taiwan (Murase 2015). Although tidal pool fishes have been fewer reported compared to more than 600 species in the adjacent waters, we think tidal pools of Jeju Island are very important for fish habitat, zoogeography, and worth of conservation.

**Table 1.** Tidal pool fishes in Jeju Island, Korea. New records in this paper indicated with a tick after the species name.

Scientific name	Distribution	Regions of occurrence	Record of Jeju Island (Kim et al. 2009)
** Clupeiformes **			
Engraulidae			
* Engraulis japonicus*	Temperate	Sindo, Yerae	√
Clupeidae			
* Spratelloides gracilis*	Tropical	Yerae	√
** Siluriformes **			
Plotosidae			
* Plotosus lineatus*	Tropical	Sindo, Seongsan	√
** Mugiliformes **			
Mugilidae			
* Chelon macrolepis*	Tropical	Yerae, Seongsan	–
* Mugil cephalus*	Subtropical	Sindo, Yerae	√
* Moolgarda seheli*	Tropical	Seongsan	–
* Oedalechilus labiosus*	Tropical	Yerae, Seongsan	–
** Atheriniformes **			
Notocheiridae			
* Iso flosmaris*√	Temperate	Sindo	√
Atherinidae			
* Atherion elymus*	Tropical	Oedo, Sindo, Yerae, Seongsan	√
** Beloniformes **			
Hemiramphidae			
* Hyporhamphus sajori*√	Temperate	Oedo	√
** Scorpaeniformes **			
Scorpaenidae			
* Sebastes inermis*√	Subtropical	Seongsan	√
* Hypodytes rubripinnis*√	Tropical	Seongsan	√
Hexagrammidae			
* Hexagrammos otakii*	Temperate	Sindo, Yerae, Seongsan	√
Cottidae			
* Furcina osimae*√	Temperate	Sindo, Seongsan	√
* Pseudoblennius cottoides*	Temperate	Seongsan	√
* Pseudoblennius percoides*√	Temperate	Seongsan	√
* Pseudoblennius marmoratus*√	Temperate	Seongsan	√
* Vellitor centropomus*√	Temperate	Seongsan	√
Cyclopteridae			
* Eumicrotremus uenoi*√	Temperate	Seongsan	–
** Perciformes **			
Plesiopidae			
* Plesiops coeruleolineatus*√	Tropical	Oedo	√
Apogonidae			
* Apogon doederleini*	Subtropical	Oedo, Yerae	√
Lutjanidae			
* Lutjanus fulviflamma*√	Tropical	Yerae	√
Gerreidae			
* Gerres oyena*√	Tropical	Yerae	√
Pempheridae			
* Pempheris japonica*√	Tropical	Sindo	√
* Pempheris schwenkii*	Tropical	Oedo, Sindo, Yerae, Seongsan	√
Kyphosidae			
* Girella leonina*	Subtropical	Yerae, Seongsan	–
* Girella punctata*	Temperate	Oedo, Sindo, Yerae, Seongsan	√
* Microcanthus strigatus*	Tropical	Oedo, Sindo, Yerae, Seongsan	√
Chaetodontidae			
* Chaetodon auriga*√	Tropical	Seongsan	√
Teraponidae			
* Terapon jarbua*	Tropical	Yerae, Seongsan	√
Kuhliidae			
* Kuhlia mugil*	Tropical	Oedo, Sindo, Yerae, Seongsan	√
Oplegnathidae			
* Oplegnathus fasciatus*	Temperate	Oedo	√
Cheilodactylidae			
* Goniistius zonatus*√	Tropical	Seongsan	√
Pomacentridae			
* Abudefduf bengalensis*	Tropical	Oedo, Sindo, Yerae	–
* Abudefduf notatus*	Tropical	Oedo, Sindo, Yerae, Seongsan	√
* Abudefduf septemfasciatus*	Tropical	Oedo, Sindo, Yerae, Seongsan	–
* Abudefduf sexfasciatus*√	Tropical	Seongsan	√
* Abudefduf sordidus*	Tropical	Oedo, Sindo, Yerae, Seongsan	√
* Abudefduf vaigiensis*	Tropical	Sindo, Yerae, Seongsan	√
* Chromis notata*	Subtropical	Oedo, Sindo, Yerae, Seongsan	√
* Pomacentrus coelestis*	Tropical	Sindo, Yerae, Seongsan	√
Labridae			
* Halichoeres poecilopterus*	Subtropical	Yerae	√
* Halichoeres tenuispinis*	Tropical	Oedo, Sindo, Yerae, Seongsan	√
* Pteragogus flagellifer*	Tropical	Sindo	√
* Stethojulis interrupta terina*√	Tropical	Seongsan	√
* Stethojulis trilineata*√	Tropical	Seongsan	–
Stichaeidae			
* Dictyosoma burgeri*	Temperate	Sindo, Seongsan	√
* Dictyosoma rubrimaculatum*	Subtropical	Seongsan	√
* Zoarchias major*	Temperate	Oedo, Sindo, Seongsan	√

* Enneapterygius etheostomus*	Temperate	Oedo, Sindo, Yerae, Seongsan	√
Blenniidae			
* Entomacrodus stellifer stellifer*	Tropical	Oedo, Yerae	√
* Istiblennius dussumieri*	Tropical	Sindo	√
* Istiblennius enosimae*	Tropical	Oedo, Sindo, Yerae, Seongsan	√
* Omobranchus elegans*	Subtropical	Oedo, Sindo, Yerae, Seongsan	√
* Parablennius yatabei*	Temperate	Sindo, Yerae, Seongsan	√
* Petroscirtes breviceps*	Tropical	Oedo, Sindo, Yerae, Seongsan	√
Chaenopsidae			
* Neoclinus bryope*	Temperate	Seongsan	√
Gobiesocidae			
* Aspasma minima*	Subtropical	Oedo, Sindo, Seongsan	–
* Lepadichthys frenatus*√	Temperate	Yerae	√
Gobiidae			
* Bathygobius fuscus*	Tropical	Oedo, Sindo, Yerae, Seongsan	√
* Chaenogobius annularis*	Temperate	Oedo, Sindo, Yerae, Seongsan	√
* Chaenogobius gulosus*	Temperate	Oedo, Sindo, Yerae, Seongsan	√
* Eviota abax*	Temperate	Oedo, Sindo, Yerae, Seongsan	√
* Eviota prasina*	Tropical	Yerae	√
* Istigobius campbelli*	Tropical	Oedo, Yerae, Seongsan	√
* Luciogobius guttatus*√	Subtropical	Oedo, Sindo, Yerae, Seongsan	√
* Clariger cosmurus*√	Temperate	Yerae	–
* Mugilogobius fontinalis*	Tropical	Sindo	√
* Tridentiger obscurus*	Temperate	Yerae	√
* Trimma grammistes*√	Temperate	Sindo	√
Ptereleotridae			
* Parioglossus dotui*	Subtropical	Oedo, Sindo, Yerae, Seongsan	√
Siganidae			
* Siganus fuscescens*√	Tropical	Oedo, Seongsan	√
** Tetraodontiformes **			
Monacanthidae			
* Stephanolepis cirrhifer*√	Temperate	Oedo, Seongsan	√
Tetraodontidae			
* Arothron hispidus*√	Tropical	Yerae, Seongsan	√
* Arothron stellatus*√	Tropical	Seongsan	√
* Takifugu niphobles*	Temperate	Yerae, Seongsan	√

**Table 2.** Number of fish species and major fish families in tidal pools in four regions of Jeju Island, Korea.

Region	No. of species	Major speciose family
Oedo (north)	31	Gobiidae (7 species) Pomacentridae (5 species) Blenniidae (4 species)
Seongsan (east)	54	Pomacentridae (7 species) Gobiidae (7 species) Cottidae (5 species)
Sindo (west)	38	Gobiidae (8 species) Pomacentridae (7 species) Blenniidae (5 species)
Yerae (south)	44	Gobiidae (10 species) Pomacentridae (7 species) Blenniidae (5 species)

In terms of distribution, most species were in the tropical group (39 species), followed by the temperate group (26) and subtropical group (11) (Table 1). Diverse and complicated water masses exist around Jeju Island (Kim et al. 2009); especially adjacent waters are known to be influenced directly by the high temperature and salinity Tsushima Warm Current, and so subtropical fishes are frequently found (Kim 2009). The present result showed the tropical and subtropical species to comprise 65.8% (50 species) of the total fish fauna, but most tropical and subtropical species have been recorded by Kim et al. (2009). Therefore, the present species diversity is not related to climate change, and continuous monitoring studies are needed to understand species dynamics.

Twenty-six species represent new records for the island, compared to the study by Kwun et al. (2017). Ten species were not listed for Korea before (cf. Kim et al. 2009). Eight species (*Hypodytes rubripinnis*, *Furcina osimae*, *Pseudoblennius percoides*, *P. marmoratus*, *Vellitor centropomus*, *Eumicrotremus uenoi*, *Goniistius zonatus*, and *Clariger cosmurus*) represent new reports of occurrence in winter and spring. Although sampling did not occur in all months, three species (*Enneapterygius etheostomus*, *Entomacrodus stellifer stellifer*, *Eviota abax*) were present in all seasons, suggesting that they are year-round residents of the tidal pools.

## Species notes

*Eumicrotremus uenoi* Kai, Ikeguchi & Nakabo, 2017 (Fig. 5B) was recorded in Korea as *Lethotremus awae* (Kim 2015), but *Lethotremus* is a junior synonym of *Eumicrotremus* and this species is reported for the new record through recent taxonomic reviewes (Lee et al. 2017, Oku et al. 2017).

The species of *Pempheris* (Fig. 7A) differed from *P. japonica* in melanophore pattern (Okiyama 2014), but a molecular comparison showed it to correspond to *P. japonica*. It may represent a difference in larvae growth.

*Girella leonina* (Richardson, 1846) was recorded in Korea as *G. melanichthys* (An 2011, Kwun et al. 2017), but *G. melanichthys* is a junior synonym of *G. leonina* (Yagishita and Nakabo 2000, Nakabo 2013).

A partial albino specimen of *Dictyosoma rubrimaculatum* Yatsu, Yasuda & Taki, 1978 was collected, representing the first report of albinism in this species (Kwun et al. 2016a).

## Appendix

Voucher specimens for tidal pool fishes obtained from Jeju Island, Korea.

***
Engraulis japonicus* Temminck & Schlegel, 1846**

Voucher: MFD-457, Yerae, 14 Oct 2015; MFD-704–706, Yerae, 14 Jun 2016; no voucher specimen from Sindo.

***
Spratelloides gracilis* (Temminck & Schlegel, 1846)**

Voucher: MFD-334–335, Yerae, 10 Sep 2015; MFD-462, Yerae, 14 Oct 2015.

***
Plotosus lineatus* (Thunberg, 1787)**

Voucher: MFD-237–243, Seongsan, 27 Aug 2015; MFD-725–731, Sindo, 31 Jul 2016; MFD-915, Sindo, 27 Oct 2016.

***
Chelon macrolepis* (Smith, 1846)**

Voucher: MFD-5–10, Seongsan, 29 Jun 2015; MFD-74, Yerae, 17 Jul 2015; MFD-209–211, Yerae, 26 Aug 2015; MFD-344, Seongsan, 9 Sep 2015; MFD-368–372, Yerae, 23 Sep 2015; MFD-410, Seongsan, 13 Oct 2015; MFD-500, Yerae, 28 Oct 2015; MFD-703, Yerae, 14 Jun 2016; MFD-783, Seongsan, 29 Jul 2016.

***
Mugil cephalus* Linnaeus, 1758**

Voucher: MFD-73, Yerae, 17 Jul 2015; MFD-193, Yerae, 26 Aug 2015; MFD-200, Sindo, 25 Aug 2015.

***
Moolgarda seheli* (Forsskål, 1775)**

Voucher: MFD-411, Seongsan, 13 Oct 2015; MFD-784, Seongsan, 29 Jul 2016.

***
Oedalechlius labiosus* (Valenciennes, 1836)**

Voucher: MFD-339, Yerae, 10 Sep 2015; MFD-343, 345, Seongsan, 9 Sep 2015; MFD-785–796, Seongsan, 29 Jul 2016.

***
Iso flosmaris* Jordan & Starks, 1901**

Voucher: MFD-876–879, Sindo, 26 Sep 2016.

***
Atherion elymus* Jordal & Starks, 1901**

Voucher: MFD-201–208, Yerae, 26 Aug 2015; MFD-321, Sindo, 8 Sep 2015; MFD-329–332, Yerae, 10 Sep 2015; MFD-452–456, Yerae, 14 Oct 2015; no voucher specimens from Oedo and Seongsan.

***
Hyporhamphus sajori* (Temminck & Schlegel, 1846)** (Fig. 2A)

Voucher: MFD-746, Oedo, 30 Jul 2016.

***
Sebastes inermis* Cuvier, 1829** (Fig. 2B)

Voucher: MFD-902, Seongsan, 27 Sep 2016.

***
Hypodytes rubripinnis* (Temminck & Schlegel, 1843)** (Fig. 3A)

Voucher: MFD-669–670, Seongsan, 18 May 2016.

***
Hexagrammos otakii* Jordan & Starks, 1895**

Voucher: MFD-516–523, Seongsan, 17 Feb 2016; MFD-533, Yerae, 18 Feb 2016; MFD-542–543, Sindo, 18 Feb 2016.

***
Furcina osimae* Jordan & Starks, 1904** (Fig. 3B)

Voucher: MFD-627–636, Sindo, 17 May 2016; MFD-680, Seongsan, 18 May 2016.

***
Pseudoblennius cottoides* (Richardson, 1848)**

Voucher: MFD-901, Seongsan, 27 Sep 2016.

***
Pseudoblennius percoides* Gunther, 1861** (Fig. 4A)

Voucher: MFD-671–672, Seongsan, 18 May 2016; MFD-926, 931, Seongsan, 12 Jan 2017.

***
Pseudoblennius marmoratus* (Döderlein, 1884)** (Fig. 4B)

Voucher: MFD-673, Seongsan, 18 May 2016; MFD-927–930, Seongsan, 12 Jan 2017.

***
Vellitor centropomus* (Richardson, 1848)** (Fig. 5A)

Voucher: MFD-933, Seongsan, 12 Jan 2017.

***
Eumicrotremus uenoi*** (Fig. 5B)

Voucher: MFD-932, Seongsan, 12 Jan 2017.

***
Plesiops coeruleolineatus* Ruppell, 1835** (Fig. 6A)

Voucher: MFD-860, Oedo, 11 Aug 2016.

***
Apogon deoderleini* Jordan & Snyder, 1901**

Voucher: MFD-319–320, Oedo, 7 Sep 2015; MFD-324, Yerae, 10 Sep 2015.

***
Lutjanus fulviflamma* (Forsskål, 1775)** (Fig. 6B)

Voucher: MFD-866, Yerae, 25 Sep 2016.

***
Gerres oyena* (Forsskål, 1775)**

Voucher: MFD-873–874, Yerae, 26 Sep 2016.

***
Pempheris japonica*** (Fig. 7A)

Voucher: MFD-894–895, Sindo, 26 Sep 2016.

***
Pempheris schwenkii* Bleeker, 1855**

Voucher: MFD-104–106, Yerae, 13 Aug 2015; MFD-345–357, Oedo, 22 Sep 2015; MFD-373–379, Yerae, 23 Sep 2015; MFD-412, Seongsan, 13 Oct 2015; MFD-430, Yerae, 14 Oct 2015; MFD-891–893, Sindo, 26 Sep 2016.

***
Girella leonina* (Richardson, 1846)**

Voucher: MFD-801, Seongsan, 29 Jul 2016.

***
Girella punctata* Gray, 1835**

Voucher: MFD-27, Seongsan, 15 Jul 2015; MFD-426, Yerae, 14 Oct 2015; MFD-619–621, Oedo, 16 May 2016; MFD-643, Sindo, 17 May 2016; MFD-676–677, Seongsan, 18 May 2016; MFD-800, Seongsan, 29 Jul 2016.

***
Microcanthus strigatus* Swainson, 1839**

Voucher: MFD-29, Seongsan, 15 Jul 2015; MFD-49–52, Sindo, 16 Jul 2015; MFD-458–461, Yerae, 14 Oct 2015; MFD-494, Sindo, 27 Oct 2015; MFD-625, Oedo, 16 May 2016; MFD-696, Yerae, 19 May 2016; MFD-780, Seongsan, 29 Jul 2016.

***
Chaetodon auriga* Forsskål, 1775** (Fig. 7B)

Voucher: MFD-778, Seongsan, 29 Jul 2016.

***
Terapon jarbua* (Forsskål, 1775)**

Voucher: MFD-95–96, Yerae, 13 Aug 2015; MFD-187–192, Yerae, 26 Aug 2015; MFD-235, Seongsan, 27 Aug 2015; MFD-406, Seongsan, 13 Oct 2015; MFD-486–489, Seongsan, 27 Oct 2015; MFD-708–711, Seongsan, 15 Jun 2016; MFD-782, Seongsan, 29 Jul 2016; MFD-857, Seongsan, 9 Aug 2016; MFD-918, Seongsan, 29 Oct 2016.

***
Kuhlia mugil* (Forster, 1829)**

Voucher: MFD-338, Yerae, 10 Sep 2015; MFD-432–434, Yerae, 14 Oct 2015; MFD-481, Oedo, 26 Oct 2015; MFD-501–502, Yerae, 28 Oct 2015; MFD-740–745, Sindo, 31 Jul 2016; MFD-758–767, Oedo, 30 Jul 2016; MFD-777, Yerae, 28 Jul 2016; MFD-867–869, Yerae, 25 Sep 2016; MFD-880–881, Sindo, 26 Sep 2016; MFD-897–898, Seongsan, 27 Sep 2016; MFD-909, Yerae, 27 Oct 2016.

***
Oplegnathus fasciatus* (Temminck & Schlegel, 1844)**

Voucher: MFD-155, Oedo, 24 Aug 2015.

***
Goniistius zonatus* (Cuvier, 1830)** (Fig. 8A)

Voucher: MFD-663, Seongsan, 18 May 2016.

***
Abudefduf bengalensis* (Bloch, 1787)**

Voucher: MFD-194, Yerae, 26 Aug 2015; MFD-757, Oedo, 30 Jul 2016; MFD-882, Sindo, 26 Sep 2016.

***
Abudefduf notatus* (Day, 1870)**

Voucher: MFD-42–43, Sindo, 16 Jul 2015; MFD-121–127, Seongsan, 14 Aug 2015; MFD-154, Oedo, 24 Aug 2015; MFD-170–173, Sindo, 25 Aug 2015; MFD-176, Yerae, 26 Aug 2015; MFD-219–230, Seongsan, 27 Aug 2015; MFD-336, Yerae, 10 Sep 2015; MFD-463, Oedo, 26 Oct 2015; MFD-756, Oedo, 30 Jul 2016; MFD- 781, Seongsan, 29 Jul 2016; MFD-870–872, Yerae, 25 Sep 2016; MFD-900, Seongsan, 27 Sep 2016.

***
Abudefduf septemfasciatus* (Cuvier, 1830)**

Voucher: MFD-196–197, Yerae, 26 Aug 2015; MFD-198, Oedo, 24 Aug 2015; MFD-218, Seongsan, 27 Aug 2015; MFD-312–317, Sindo, 8 Sep 2015; MFD-347–349, Oedo, 22 Sep 2015; MFD-911, Yerae, 27 Oct 2016.

***
Abudefduf sexfasciatus* (Lacepède, 1801)** (Fig. 8B)

Voucher: MFD-845, Seongsan, 9 Aug 2016.

***
Abudefduf sordidus* (Forsskål, 1775)**

Voucher: MFD-3–4, Seongsan, 29 Jun 2015; MFD-11, Sindo, 29 Jun 2015; MFD-48, Sindo, 16 Jul 2015; MFD-79, Yerae, 17 Jul 2015; MFD-120, Yerae, 13 Aug 2015; MFD-195, Yerae, 26 Aug 2015; MFD-199, Oedo, 24 Aug 2015; MFD-337, Yerae, 10 Sep 2015; MFD-340–341, Seongsan, 9 Sep 2015; MFD-350–351, Oedo, 22 Sep 2015; MFD-490, Sindo, 27 Oct 2015; MFD-503–508, Yerae, 28 Oct 2015; MFD-753–754, Oedo, 30 Jul 2016; MFD-776, Yerae, 28 Jul 2016; MFD-899, Seongsan, 27 Sep 2016; MFD-910, Yerae, 27 Oct 2016.

***
Abudefduf vaigiensis* (Quoy & Gaimard, 1825)**

Voucher: MFD-76, Yerae, 17 Jul 2015; MFD-118–119, Yerae, 13 Aug 2015; MFD-342, Seongsan, 9 Sep 2015; MFD-409, Seongsan, 13 Oct 2015; MFD-484, Seongsan. 27 Oct 2015; MFD-491, Sindo, 27 Oct 2015; MFD-707, Seongsan, 15 Jun 2016; MFD-779, Seongsan, 29 Jul 2016; MFD-852–854, Seongsan, 9 Aug 2016.

***
Chromis notata* (Temminck & Schlegel, 1843)**

Voucher: MFD-93, Yerae, 13 Aug 2015; MFD-134–135, Oedo, 14 Aug 2015; MFD-146, Oedo, 24 Aug 2015; MFD-185–186, Yerae, 26 Aug 2015; MFD-311, Sindo, 8 Sep 2015; MFD-322–323, Yerae, 10 Sep 2015; MFD-414, Seongsan, 13 Oct 2015; MFD-435–445, Yerae, 14 Oct 2015; MFD-499, Yerae, 28 Oct 2015; MFD-827, 846–848, Seongsan, 9 Aug 2016.

***
Pomacentrus coelestis* Jordan & Starks, 1901**

Voucher: MFD-396, Sindo, 15 Oct 2015; MFD-415, Seongsan, 13 Oct 2015; MFD-492–493, Sindo, 27 Oct 2015; MFD-908, Yerae, 27 Oct 2016; MFD-912, Sindo, 27 Oct 2016.

***
Halichoeres poecilopterus* (Temminck & Schlegel, 1845)**

Voucher: no voucher specimen from Yerae.

***
Halichoeres tenuispinis* (Gunther, 1862)**

Voucher: MFD-129, Oedo, 14 Aug 2015; MFD-149–151, Oedo, 24 Aug 2015; MFD-212–214, Oedo, 14 Aug 2015; MFD-295–296, Oedo, 7 Sep 2015; MFD-310, Sindo, 8 Sep 2015; MFD-325–326, Yerae, 10 Sep 2015; MFD-346, Seongsan, 9 Sep 2015; MFD-355, Oedo, 22 Sep 2015; MFD-366–367, Yerae, 23 Sep 2015; MFD-402–403, Seongsan, 13 Oct 2015; MFD-424–425, Yerae, 14 Oct 2015; MFD-476, Oedo, 26 Oct 2015; MFD-695, Yerae, 19 May 2016; MFD-829–831, erae, 10 Aug 2016; MFD-883, Sindo, 26 Sep 2016.

***
Pteragogus flagellifer* (Valenciennes, 1839)**

Voucher: MFD-397, Sindo, 15 Oct 2015; MFD-875, Sindo, 26 Sep 2016.

***
Stethojulis interrupta terina* Jordan & Snyder, 1902** (Fig. 9A)

Voucher: MFD-850, Seongsan, 9 Aug 2016.

***
Stethojulis trilineata* (Bloch & Schneider, 1801)** (Fig. 9B)

Voucher: MFD-482, Seongsan, 27 Oct 2015.

***
Dictyosoma burgeri* van der Hoeven, 1855**

Voucher: MFD-58–59, Seongsan, 15 Jul 2015; MFD-394, Sindo, 15 Oct 2015; MFD-512, Seongsan, 14 Aug 2015; MFD-659–660, Seongsan, 18 May 2016.

***
Dictyosoma rubrimaculatum* Yatsu, Yasuda & Taki, 1978**

Voucher: MFD-57, Albino, Seongsan, 15 Jul 2015; MFD-244, Seongsan, 27 Aug 2015; MFD-485, Seongsan, 27 Oct 2015; MFD-661–662, Seongsan, 18 May 2016; MFD-916, Albino, Seongsan, 29 Oct 2016.

***
Zoarchias major* Tomiyama, 1972**

Voucher: MFD-41, Oedo, 14 Jul 2015; MFD-60, Seongsan, 15 Jul 2015; MFD-524–531, Seongsan, 17 Feb 2016; MFD-616–618, Oedo, 16 May 2016; MFD-646, Sindo, 17 May 2016; MFD-664–667, Seongsan, 18 May 2016; MFD-919–923, Seongsan, 12 Jan 2017.

***
Enneapterygius etheostomus* (Jordan & Snyder, 1902)**

Voucher: MFD-16, Oedo, 29 Jun 2015; MFD-40, Oedo, 14 Jul 2015; MFD-308, Sindo, 8 Sep 2015; MFD-423, Yerae, 14 Oct 2015; MFD-534–538, Yerae, 18 Feb 2016; MFD-544, Sindo, 18 Feb 2016; MFD-668, Seongsan, 18 May 2016.

***
Entomacrodus stellifer stellifer* (Jordan & Snyder, 1902)**

Voucher: MFD-80, Yerae, 17 Jul 2015; MFD-175, Yerae, 26 Aug 2015; MFD-428, Yerae, 14 Oct 2015; MFD-496, Yerae, 28 Oct 2015; MFD-539–540, Yerae, 18 Feb 2016; MFD-698–700, Yerae, 19 May 2016; no voucher specimen from Oedo.

***
Istiblennius dussumieri* (Valenciennes, 1836)**

Voucher: MFD-309, Sindo, 8 Sep 2015.

***
Istiblennius enosimae* (Jordan & Snyder, 1902)**

Voucher: MFD-380, Oedo, 13 Oct 2015; MFD-407, Seongsan, 13 Oct 2015; MFD-427, Yerae, 14 Oct 2015; MFD-532, Yerae, 18 Feb 2016; no voucher specimen from Sindo.

***
Omobranchus elegans* (Steindachner, 1876)**

Voucher: MFD-81, Yerae, 17 Jul 2015; MFD-91, Seongsan, 12 Aug 2015; MFD-97, Yerae, 13 Aug 2015; MFD-156–157, Oedo, 24 Aug 2015; MFD-328, Yerae, 10 Sep 2015; MFD-352, Oedo, 22 Sep 2015; MFD-404, Seongsan, 13 Oct 2015; MFD-681, Seongsan, 18 May 2016; MFD-751, Oedo, 30 Jul 2016; no voucher specimen from Sindo.

***
Parablennius yatabei* (Jordan & Snyder, 1900)**

Voucher: MFD-429, Yerae, 14 Oct 2015; MFD-513, Seongsan, 14 Aug 2015; MFD-626, Sindo, 17 May 2016; MFD-697, Yerae, 19 May 2016; MFD-797, Seongsan, 29 Jul 2016.

***
Petroscirtes breviceps* (Valenciennes, 1836)**

Voucher: MFD-92, Yerae, 13 Aug 2015; MFD-161, Sindo, 25 Aug 2015; MFD-297–302, Oedo, 7 Sep 2015; MFD-327, Yerae, 10 Sep 2015; MFD-405, Seongsan, 13 Oct 2015; MFD-737–139, Sindo, 31 Jul 2016; MFD-755, Oedo, 30 Jul 2016; MFD-802–806, Seongsan, 29 Jul 2016.

***
Neoclinus bryope* (Jordan & Snyder, 1902)**

Voucher: MFD-53, Seongsan, 15 Jul 2015; MFD-798–799, Seongsan, 29 Jul 2016.

***
Aspasma minima* (Döderlein, 1887)**

Voucher: MFD-389–390, Oedo, 13 Oct 2015; MFD-413, Seongsan, 13 Oct 2015; MFD-477–480, Oedo, 26 Oct 2015; MFD-483, Seongsan, 27 Oct 2015; MFD-888–890, Sindo, 26 Sep 2016; MFD-924–925, Seongsan, 12 Jan 2017.

***
Lepadichthys frenatus* Waite, 1904** (Fig. 10A)

Voucher: MFD-318, Yerae, 17 Aug 2015.

***
Bathygobius fuscus* (Ruppell, 1830)**

Voucher: MFD-54–56, Seongsan, 15 Jul 2015; MFD-78, Yerae, 17 Jul 2015; MFD-398–399, Seongsan, 13 Oct 2015; MFD-416, Yerae, 14 Oct 2015; MFD-674–675, Seongsan, 18 May 2016; MFD-748, Oedo, 30 Jul 2016; MFD-896, Sindo, 26 Sep 2016; MFD-906–907, Oedo, 28 Oct 2016.

***
Chaenogobius annularis* Gill, 1859**

Voucher: MFD-12, Sindo, 29 Jun 2015; MFD-28, Oedo, 14 Jul 2015; MFD-648, Sindo, 17 May 2016; MFD-732–933, Sindo, 31 Jul 2016; MFD-747, Oedo, 30 Jul 2016; no voucher specimens from Yerae and Seongsan.

***
Chaenogobius gulosus* (Sauvage, 1882)**

Voucher: MFD-20, Oedo, 14 Jul 2015; MFD-774–775, Yerae, 28 Jul 2016; no voucher specimens from Sindo and Seongsan.

***
Eviota abax* (Jordan & Snyder, 1901)**

Voucher: MFD-19, Yerae, 30 Jun 2015; MFD-30–33, Seongsan, 15 Jul 2015; MFD-75, Yerae, 17 Jul 2015; MFD-136–142. Seongsan, 14 Aug 2015; MFD-231–233, Seongsan, 27 Aug 2015; MFD-288–289, Oedo, 7 Sep 2015; MFD-381–384, Oedo, 13 Oct 2015; MFD-395, Sindo, 15 Oct 2015; MFD-401, Seongsan, 13 Oct 2015; MFD-464–472, Oedo, 26 Oct 2015; MFD-541, Yerae, 18 Feb 2016; MFD-624, Oedo, 16 May 2016; MFD-644–645, Sindo, 17 May 2016; MFD-678–679, Seongsan, 18 May 2016; MFD-689–694, Yerae, 19 May 2016; MFD-749–750, Oedo, 30 Jul 2016; MFD-772–773, Yerae, 28 Jul 2016; MFD-913–914, Sindo, 27 Oct 2016; MFD-917, Seongsan, 29 Oct 2016.

***
Eviota prasina* (Klunzinger, 1871)**

Voucher: MFD-77, Yerae, 17 Jul 2015; MFD-183–184, Yerae, 26 Aug 2015; MFD-431, Yerae, 14 Oct 2015; MFD-828, Yerae, 10 Aug 2016.

***
Istigobius campbelli* (Jordan & Snyder, 1901)**

Voucher: MFD-131–133, Oedo, 14 Aug 2015; MFD-147–148, Oedo, 24 Aug 2015; MFD-234, Seongsan, 27 Aug 2015; MFD-333, Yerae, 10 Sep 2015; MFD-353–354, Oedo, 22 Sep 2015; MFD-385–388, Oedo, 13 Oct 2015; MFD-400, Seongsan, 13 Oct 2015; MFD-422, Yerae, 14 Oct 2015; MFD-473–475, Oedo, 26 Oct 2015; MFD-771, Yerae, 28 Jul 2016.

***
Luciogobius guttatus* Gill, 1859** (Fig. 10B)

Voucher: MFD-130, Oedo, 14 Aug 2015; MFD-144–145, Seongsan, 14 Aug 2015; MFD-545, Sindo, 18 Feb 2016; no voucher specimen from Yerae.

***
Clariger cosmurus* Jordan & Snyder, 1901** (Fig. 11A)

Voucher: MFD-682–683, Yerae, 19 May 2016.

***
Mugilogobius fontinalis* (Jordan & Seale, 1906)**

Voucher: MFD-17–18, Sindo, 29 Jun 2015; MFD-61–72, Sindo, 16 Jul 2015; MFD-98–103, Sindo, 13 Aug 2015; MFD-162×169, Sindo, 25 Aug 2015; MFD-303–307, Sindo, 8 Sep 2015; MFD-391–393, Sindo, 15 Oct 2015; MFD-637–642, Sindo, 17 May 2016; MFD-734, Sindo, 31 Jul 2016; MFD-884–887, Sindo, 26 Sep 2016.

***
Tridentiger obscurus* (Temminck & Schlegel, 1845)**

Voucher: MFD-417–421, Yerae, 14 Oct 2015; MFD-684, Yerae, 19 May 2016; MFD-769–770, Yerae, 28 Jul 2016.

***
Trimma grammistes* (Tomiyama, 1936)** (Fig. 11B)

Voucher: MFD-495, Sindo, 27 Oct 2015.

***
Parioglossus dotui* Tomiyama, 1958**

Voucher: MFD-13, Oedo, 29 Jun 2015; MFD-34–39, Oedo, 14 Jul 2015; MFD-82–83, Yerae, 17 Jul 2015; MFD-143, Oedo, 14 Aug 2015; MFD-152–153, Oedo, 24 Aug 2015; MFD-158–160, Sindo, 24 Aug 2015; MFD-177–182, Yerae, 26 Aug 2015; MFD-290–294, Oedo, 7 Sep 2015; MFD-358–365, Oedo, 22 Sep 2015; MFD-446–451, Yerae, 14 Oct 2015; MFD-497–498, Yerae, 28 Oct 2015; MFD-622–623, Oedo, 16 May 2016; MFD-647, Sindo, 17 May 2016; MFD-685–688, Yerae, 19 May 2016; MFD-735–736, Sindo, 31 Jul 2016; MFD-752, Oedo, 30 Jul 2016; no voucher specimen from Seongsan.

***
Siganus fuscescens* (Houttuyn, 1782)**

Voucher: MFD-122, Seongsan, 14 Aug 2015; MFD-832, Oedo, 11 Aug 2016.

***
Stephanolepis cirrhifer* (Temminck & Schlegel, 1850)**

Voucher: MFD-128, Oedo, 14 Aug 2015; MFD-858–859, Seongsan, 9 Aug 2016.

***
Arothron hispidus* (Linnaeus, 1758)** (Fig. 12A)

Voucher: MFD-768, Yerae, 28 Jul 2016; MFD-807, Seongsan, 29 Jul 2016; MFD-851, Seongsan, 9 Aug 2016.

***
Arothron stellatus* (Anonymous, 1798)** (Fig. 12B)

Voucher: MFD-808, Seongsan, 29 Jul 2016.

***
Takifugu niphobles* (Jordan & Snyder, 1901)**

Voucher: MFD-174, Yerae, 26 Aug 2015; MFD-236, Seongsan, 27 Aug 2015; MFD-408, Seongsan, 13 Oct 2015.
